# Symptoms and Factors Associated With Eustachian Tube Dysfunction Among the Population of Qassim, Saudi Arabia

**DOI:** 10.7759/cureus.61087

**Published:** 2024-05-26

**Authors:** Abdulhakeem Almutairi, Bassam A Alharbi, Mohammed T Alharbi, Abdulrahman F Al-Sowinea, Atheer M Alshammakhi, Fadi A Munhish, Anad M Al-Harbi, Jehad M Alabdulrahim

**Affiliations:** 1 Department of Otolaryngology, Head and Neck Surgery, Qassim University, Buraydah, SAU; 2 College of Medicine, Qassim University, Buraydah, SAU; 3 College of Medicine, Jazan University, Jazan, SAU

**Keywords:** aural fullness, ear pressure, tinnitus, otalgia, eustachian tube dysfunction

## Abstract

Introduction

The Eustachian tube regulates middle ear functions such as ventilation and pressure normalization. Eustachian tube dysfunction (ETD) is defined as the failure of the Eustachian tube to maintain one or more of its functions. It is a common condition that is associated with other middle ear disorders such as cholesteatoma, tympanic membrane atelectasis, and otitis media with effusion (OME). This study aims to assess ETD prevalence and risk factors in the Qassim region.

Methodology

This cross-sectional study was conducted in the Qassim region of Saudi Arabia during the period from September 20 to October 10, 2023. Data were gathered via a validated, self-administered electronic questionnaire that encompasses socio-demographic information, the prevalence of ETD, and the prevalence of its various symptoms, as assessed by the Eustachian Tube Dysfunction Questionnaire (ETDQ-7).

Results

Our study in Qassim, Saudi Arabia, with 467 participants reveals a high prevalence of ETD at 12.2%. The 18-25 age group dominates (50.1%), with a majority of females (66.2%). Symptom analysis using the ETDQ-7 questionnaire shows varied prevalence, with severe pain (7%) and muffled hearing (29.8%) notable. Logistic regression identifies significant predictors, including hearing loss history (odds ratio = 28.2) and smoking (odds ratio = 3.70). Specific symptoms, such as feeling blocked or underwater, significantly correlate with more severe ETD symptoms (odds ratio = 1.73).

Conclusion

Our study highlights a notable prevalence of ETD. Significant predictors, including hearing loss history and smoking, were identified. Specific symptoms, such as feeling blocked or underwater, were associated with more severe ETD symptoms.

## Introduction

The Eustachian tube, or auditory tube, is a vital part of the middle ear structures that connect the middle ear cavity medially with the nasopharynx [1]. It carries out many functions that regulate middle ear homeostasis, including pressure equalization, ventilation of the middle ear cavity, protection from loud sounds, and mucociliary clearance [2, 3]. Additionally, it prevents nasopharyngeal sound and fluid reflux [2, 4, 5]. Eustachian tube dysfunction (ETD) is defined as the failure of the Eustachian tube to maintain one or more of its functions [2]. It is a common condition that is associated with other middle ear disorders such as cholesteatoma, tympanic membrane atelectasis, and otitis media with effusion (OME) [1, 2], which could cause hearing difficulty and delayed speech in children [2]. Moreover, long-term ETD can affect patients’ quality of life and productivity [6].

Eustachian tube dysfunction and related diseases are linked to two million clinic visits annually among patients over the age of 20 and two million, 600,000 visits among those under the age of 20 [7]. Many studies have found that up to 70% of patients undergoing tympanoplasty for middle ear diseases such as cholesteatoma and chronic otitis media had ETD [8-9]. However, a recent study performed in Taif, Saudi Arabia, with 693 participants showed that about one-fifth of the participants (21.1%) had ETD [10].

Eustachian tube dysfunction patients may present with symptoms of pressure disequilibrium, including discomfort, pain, or ‘popping’ [6, 10]. Other symptoms include an ‘underwater’ sensation, crackling, ringing, or hearing loss [3]. Patients may report a history of allergic rhinitis, viral upper respiratory tract infection, or otitis media [3, 5, 6, 10-12]. It has been challenging to find a single gold standard method to evaluate the Eustachian tube function, as the current way requires several tools and trained staff, which are mostly available in specialized centers [6, 13]. Therefore, a simple subjective instrument (the Eustachian Tube Dysfunction Questionnaire (ETDQ-7)) is used as a self-report instrument to evaluate the symptoms associated with ETD and the outcomes of the treatment [14].

Knowledge of disease prevalence in the population can help determine existing and future community service needs. To the best of our knowledge, no research into the prevalence of ETD in this community has been conducted in Saudi Arabia. Hence, the goal of this study is to determine the prevalence of ETD and risk factors in the population of Qassim, which will be of value in establishing possible current and future community service requirements.

## Materials and methods

A cross-sectional study was conducted in Qassim, Saudi Arabia, to assess the prevalence of and factors associated with ETD among the general public. From September 20 to October 10, 2023, the participants were recruited randomly from the population of Qassim through the utilization of a self-administered electronic questionnaire, which was disseminated via online platforms and through direct online communication channels. A total of 467 participants were recruited from the population of Qassim city, Saudi Arabia, who agreed to participate in the study, while participants from other cities, those living outside of Qassim city, under 18 years of age, and those who refused to participate were excluded.

Data collection instrument

A validated questionnaire sourced from a previously published study was employed in the research [15]. The questionnaire was written in Arabic and included information such as the participant's gender, age, city, whether the participant has been diagnosed with hearing loss or ETD, and family history of hearing loss. It also included eight questions about the symptoms of hearing loss using the ETDQ-7, which was created by McCoul et al. [14] as a new scoring system for evaluating the symptoms associated with obstructive ETD. Each of the seven items has a score ranging from one to seven, resulting in a total composite mean score of between one and seven points when the overall score is divided by seven. A mean score of ≥2.5 is considered abnormal, with higher scores indicating a greater level of symptom severity [13]. The questionnaire was sent to the participants through various online platforms and direct online communication channels.

Ethical considerations

Before commencing data collection, authorization was acquired from the Committee of Research Ethics at the Deanship of Scientific Research, Qassim University, Buraydah, Saudi Arabia (approval number: 23-61-15). A close inspection of the research protocol and procedures was conducted to ensure compliance with ethical norms, thereby protecting the rights and welfare of participants. All participants provided informed consent to ensure their voluntary involvement. Strict measures were implemented to maintain the confidentiality and anonymity of participants' responses during both data collection and analysis.

Data analysis

The data were categorized in a Microsoft Excel sheet (Microsoft Corp., Redmond, WA) before being transferred to IBM Statistical Package for Social Sciences (SPSS) Software for Windows, version 24 (IBM Corp., Armonk, NY). A frequency table was used to describe categorical variables, while the mean and standard deviation were used to describe continuous variables. The chi-square test was used to determine the statistical significance of the data. P-values <0.05 were considered significant for all statistical tests.

## Results

Our research comprised a cohort of 467 participants, and by meticulously exploring their demographic attributes, a predominant age group ranging from 18 to 25 years emerged, constituting the largest segment (n = 234, 50.1%). Furthermore, our analysis revealed a striking predominance of females, encompassing 66.2% (n = 309) of the participants, alongside a significant representation of Saudi nationals, comprising 94.6% of the cohort (n = 442). Additionally, our investigation documented notable occurrences of hearing loss, familial predisposition to hearing impairment, and smoking habits, reported by 6.2% (n = 29), 26.8% (n = 125), and 10.9% (n = 51) of the participants, respectively. Regarding ETD, our findings indicate a prevalence rate of 12.2% among the participants (n = 57). A comprehensive overview of the data is presented in Table 1.

**Table 1:** Sociodemographic characteristics and the prevalence of ETD according to the ETDQ-7 ETD: Eustachian tube dysfunction; ETDQ-7: Eustachian Tube Dysfunction Questionnaire

		N (467)	Percentage (%)
Age (years)	18-25	234	50.1
	26-35	130	27.8
	36-45	69	14.8
	>45	34	7.3
Gender	Female	309	66.2
	Male	158	33.8
Nationality	Non-Saudi	25	5.4
	Saudi	442	94.6
Diagnosed with ETD	No	410	87.8
	Yes	57	12.2
Diagnosed with hearing loss	No	438	93.8
	Yes	29	6.2
Family history of hearing loss	No	342	73.2
	Yes	125	26.8
Smoking history	No	416	89.1
	Yes	51	10.9

**Figure 1:**
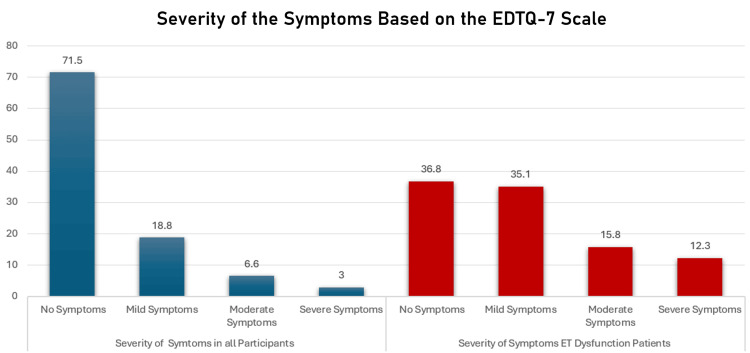
Prevalence of severity of symptoms among the participants based on the ETDQ-7 All participants (N=467); ET dysfunction patients (N=57, 12.2%) ET: Eustachian tube; ETDQ-7: Eustachian Tube Dysfunction Questionnaire

Concerning the prevalence and severity of ETD symptoms, as assessed through the ETDQ-7, within the overall population, a substantial 71.5% (n = 334) reported an absence of symptoms. Conversely, 18.8% (n = 88), 6.6% (n = 31), and 3% (n = 14) reported experiencing mild, moderate, and severe symptoms, respectively. Among individuals diagnosed with ETD (n = 57, 12.2%), a nuanced distribution of symptom severity emerged which was as follows: 36.8% (n = 21) exhibited minimal or no symptoms, 35.1% (n = 20) reported mild symptoms, 15.8% (n = 9) experienced moderate symptoms, and 12.3% (n = 7) indicated severe symptoms. By analyzing the symptoms reported by participants diagnosed with ETD according to severity, mild hearing of crackling sounds in the ears was the most frequently reported symptom, affecting 38.6% of individuals (n = 22). Notably, a moderate feeling of blocked ears or a sensation akin to being 'underwater' was reported by 21.1% (n = 12), while severe muffled hearing was reported by 29.8% (n = 17) of the participants. A graphical representation and detailed tabulation of the data are presented in Figure 1 and Table 2.

**Table 2:** The prevalence of different symptoms in the participants diagnosed with ETD according to the ETDQ-7 N= 57/467 (12.2%) ETD: Eustachian tube dysfunction; ETDQ-7: Eustachian Tube Dysfunction Questionnaire

		Minimal or no symptoms	Mild symptoms	Moderate symptoms	Severe symptoms
Feeling pressure in the ear	N	25	21	6	5
	%	43.9	36.8	10.5	8.8
Feeling pain in the ear	N	35	9	9	4
	%	61.4	15.8	15.8	7.0
The feeling that ears are blocked or 'underwater'	N	20	18	12	7
	%	35.1	31.6	21.1	12.3
The appearance of ear symptoms when catching a cold or sinus infection	N	22	16	9	10
	%	38.6	28.1	15.8	17.5
Hearing crackling sounds in the ears	N	24	22	6	5
	%	42.1	38.6	10.5	8.8
Hearing ringing in the ears	N	24	17	10	6
	%	42.1	29.8	17.5	10.5
The feeling that hearing is muffled	N	19	15	6	17
	%	33.3	26.3	10.5	29.8

Table 3 presents the outcomes of a logistic regression model elucidating adjusted predictors of ETD. Notably, age categories spanning 26-35, 36-45, and over 45 years exhibited no discernible association with ETD when compared to the reference group of 18-25 years. While gender (male) and Saudi nationality displayed non-significant p-values, they presented elevated odds ratios of 1.811 and 2.457, respectively. Nevertheless, individuals with a history of hearing loss demonstrated a significant association with ETD, boasting an odds ratio of 28.211 (95% CI: 10.973-72.528, p < 0.001). Similarly, smoking history was significantly linked with heightened odds of ETD, with an odds ratio of 3.702 (95% CI: 1.515-9.047, p = 0.004).

**Table 3:** Adjusted predictors of ETD N= 467; logistic regression model The p-value is considered significant if it is <0.05 ETD: Eustachian tube dysfunction

	B	Sig.	Exp(B)	95% CI	
				Lower	Upper
Age (18-25 years)		0.896			
Age (26-35 years)	-0.247	0.548	0.781	0.349	1.748
Age (36-45 years)	-0.086	0.860	0.917	0.352	2.388
Age (>45 years)	0.199	0.751	1.221	0.356	4.185
Gender (male)	0.594	0.122	1.811	0.852	3.846
Nationality (Saudi)	0.899	0.344	2.457	0.382	15.797
Ever been diagnosed with hearing loss	3.340	0.000	28.211	10.973	72.528
Family history of hearing loss	0.314	0.390	1.369	0.669	2.802
Smoking history	1.309	0.004	3.702	1.515	9.047

Table 4 provides insights from a logistic regression model investigating adjusted sociodemographic predictors of moderate to severe symptoms among participants diagnosed with ETD. Age emerged as a significant factor, with individuals aged 26-35 exhibiting a noteworthy increase in the odds of experiencing moderate to severe symptoms of ETD (Exp(B) = 3.899, p = 0.001), while those aged 36-45 showed a marginally significant increase (Exp(B) = 2.626, p = 0.053), in comparison to the reference group (18-25 years). Gender (male) did not emerge as a significant predictor (p = 0.155), though Saudi nationality indicated elevated odds, albeit lacking statistical significance (Exp(B) = 4.384, p = 0.197). Individuals ever diagnosed with hearing loss exhibited significantly heightened odds of experiencing moderate to severe symptoms of ETD (Exp(B) = 7.106, p <0.001), as did those with a family history of hearing loss (Exp(B) = 2.112, p = 0.033). Furthermore, a history of smoking significantly predicted moderate to severe symptoms of ETD (Exp(B) = 3.481, p = 0.016).

**Table 4:** Adjusted sociodemographic predictors of moderate-to-severe symptoms in the participants diagnosed with ETD N= 57/467 (12.2%); logistic regression model The p-value is considered significant if it is <0.05 ETD: Eustachian tube dysfunction

	B	Sig.	Exp(B)	95% CI	
				Lower	Upper
Age (18-25 years)		0.011			
Age (26-35 years)	1.361	0.001	3.899	1.721	8.832
Age (36-45 years)	0.965	0.053	2.626	0.986	6.990
Age (>45 years)	0.389	0.569	1.475	0.387	5.631
Gender (male)	-0.639	0.155	0.528	0.219	1.273
Nationality (Saudi)	1.478	0.197	4.384	0.465	41.356
Ever been diagnosed with hearing loss	1.961	0.000	7.106	2.820	17.905
Family history of hearing loss	0.748	0.033	2.112	1.061	4.205
Smoking history	1.247	0.016	3.481	1.266	9.571

Table 5 highlights specific symptoms that emerge as clinically significant predictors of moderate-to-severe symptoms among participants diagnosed with ETD. Notably, the sensation of ears being blocked or feeling 'underwater' (Exp(B) = 1.736) and experiencing symptoms during cold or sinus infections (Exp(B) = 2.340) significantly elevated the likelihood of more severe symptoms. Furthermore, hearing crackling sounds (Exp(B) = 1.827) and reporting a muffled hearing sensation (Exp(B) = 1.703) were also correlated with higher odds of experiencing moderate to severe symptoms.

**Table 5:** Adjusted odds ratio of other predictors of moderate to severe symptoms in the participants diagnosed with ETD N= 57/467 (12.2%); logistic regression model The P-value is considered significant if it is <0.05 ETD: Eustachian tube dysfunction

	B	Sig.	Exp(B)	95% CI	
				Lower	Upper
Feeling pressure in the ear	0.470	0.173	1.600	0.813	3.146
Feeling pain in the ear	0.435	0.086	1.544	0.940	2.538
The feeling that ears are blocked or 'underwater'	0.551	0.046	1.736	1.010	2.982
The appearance of ear symptoms when catching a cold or sinus infection	0.850	0.002	2.340	1.362	4.019
Hearing crackling sounds in the ears	0.603	0.012	1.827	1.142	2.924
Hearing ringing in the ears	0.318	0.226	1.374	0.822	2.299
The feeling that hearing is muffled	0.533	0.023	1.703	1.077	2.694

## Discussion

Preserving optimal middle ear function is paramount for sustaining typical auditory capabilities, necessitating the regulation of middle ear pressure to approximate ambient levels, a pivotal role orchestrated by the Eustachian tube. Consequently, our investigation endeavors to elucidate the prevalence, correlated factors, and symptomatic manifestations of ETD, given its intertwined association with middle ear pathologies [1, 2].

The aggregate prevalence of ETD in our investigation stood at 12.2%, with muffled hearing emerging as the most commonly cited symptom. This figure denotes a marginally lower prevalence in contrast to findings from local studies conducted across various regions of Saudi Arabia, which reported prevalence rates of 21.2% in Taif [10], 42.49% in Jeddah [15], and 41.3% in Al-Madinah [16]. The discerned variance could plausibly be attributed to discrepancies in sample sizes and environmental influences, given that these regions exhibit distinct behavioral patterns and cultural contexts compared to Qassim. Globally, estimates of ETD prevalence among the populations of the United Kingdom and the United States stand at 0.9% [17] and 4.6% [18], respectively.

In our investigation, logistic regression analysis has discerned hearing impairment and tobacco consumption as conceivable prognosticators of ETD. Conversely, variables such as age, gender, family history of hearing problems, and additional covariates exhibited an insignificant correlation with ETD manifestation. As delineated by Alshamani et al., the historical incidence of hearing impairment and smoking habits exhibited a statistically insignificant linkage with ETD occurrence [16]. This phenomenon may be explicated by the variegated cultural milieu and disparate environmental risk determinants endemic to Saudi Arabia, a nation characterized by expansive territorial dimensions and a heterogeneous populace.

In the realm of gender-based susceptibilities to ETD, the male gender exhibited a statistically insignificant association, consistent with the prevailing literature indicating a reduced risk among males. Alshehri et al. underscored the significance of female gender as a discernible risk factor for ETD development (p =.01), positing that disparate societal norms and behaviors between genders in Saudi Arabia may engender variance in associated risk factors [15]. Similarly, a study conducted in Brazil corroborated heightened susceptibility among females [19]. Surprisingly, Shan et al. reported that the prevalence of ETD in the US was higher in males, with a prevalence of 5.28% (95% CI 4.14-6.41) [18]. While our findings reiterate the diminished risk among males, it is imperative to acknowledge the potential influence of response bias, particularly with a predominance of female respondents, potentially skewing the observed prevalence rates among males in our study.

Unexpectedly, specific symptoms have emerged as notable indicators, prognosticating a more pronounced trajectory of ETD among afflicted individuals. These encompass the feeling that your ears are blocked or ‘underwater’, the appearance of ear symptoms during a cold or sinus infection, the hearing of crackling sounds, and muffled hearing. Correspondingly, Alshamani et al. elucidated that the paramount average score was attributed to the manifestation of ear-related symptoms during episodes of cold or sinus infections [16]. This phenomenon may be attributed to the exacerbating effects of such infections on the course of ETD. Other variables, to the best of our knowledge, have not garnered recognition as significant predictors of a more severe disease trajectory, underscoring the imperative for further investigations elucidating the clinical indicators that delineate a more severe course in ETD patients.

Furthermore, specific demographic attributes have exhibited significant associations with a heightened disease severity profile, encompassing age cohorts spanning from 18 to 35 years, individuals with a history of diagnosed hearing impairment, those with familial predispositions to hearing loss, and individuals with a smoking history. To the best of our knowledge, these findings have not previously been identified as substantive predictors of a more severe course of ETD. Nevertheless, they have been recognized as substantial risk factors predisposing individuals to ETD onset [15, 16].

Limitations

While this study contributes valuable insights, certain limitations should be acknowledged. The cross-sectional design precludes the establishment of causal relationships, and recall bias may affect the accuracy of self-reported information. Additionally, a limitation lies in the descriptive nature of the online questionnaire, which potentially excludes segments of the community lacking education, internet access, or smartphone availability, thereby potentially compromising the sample's representativeness and the generalizability of outcomes. Also, the generalizability of the findings may be influenced by the specific demographic characteristics of the study population.

Clinical implications and future directions

Our study's results carry several clinical implications. The identification of predictors and symptom profiles can inform risk stratification and guide personalized management strategies. Clinicians should pay particular attention to individuals with a history of hearing loss, smoking, and specific symptomatology, tailoring interventions to address individual needs.

Future research should delve into the molecular and genetic aspects of ETD to unravel potential underlying mechanisms. Longitudinal studies can provide insights into the natural progression of ETD and the factors influencing symptom dynamics over time. Additionally, investigating the impact of lifestyle modifications and targeted interventions on symptom alleviation and disease progression is crucial for refining treatment approaches.

## Conclusions

Our study sheds light on the prevalence, associated factors, and symptomatology of ETD in the context of Qassim, Saudi Arabia. The diverse demographic patterns, symptom profiles, and predictors identified contribute to a nuanced understanding of ETD's clinical landscape. These findings pave the way for targeted interventions, personalized management, and avenues for further research to advance our comprehension of this prevalent otologic condition. According to our prevalence findings, ETD emerges as a notable condition, albeit with a prevalence marginally lower than that reported in existing literature, underscoring the imperative for heightened attention, awareness, and further inquiry, ideally with larger sample sizes.
